# A Case Report of Whitmore's Disease: A True Masquerader

**DOI:** 10.7759/cureus.52409

**Published:** 2024-01-16

**Authors:** Chandrashekar Patil, Nikhitha Mangalagiri, Prakash Ajmera, Prashanth Kumar KS, Haritha Reddy

**Affiliations:** 1 Radiology, Mallareddy Medical College for Women, Hyderabad, IND; 2 Cardiology, Malla Reddy Narayana Multispeciality Hospital, Hyderabad, IND; 3 Radiodiagnosis, Mallareddy Medical College for Women, Hyderabad, IND; 4 Pulmonology and Intensive Care Unit, Apollo Hospital, Chennai, IND

**Keywords:** retrospective diagnosis, septicemia, cavitatory lung nodules, thoracic melioidosis, burkholderia pseudomallei

## Abstract

Melioidosis is an uncommon bacterial infection that is endemic to countries like Southeast Asia and Northern Australia but less common in temperate zones than when seen in returned travelers. This disease can affect almost every organ, with the lung being the most common organ to be involved. Here, we present a 21-year-old diabetic male who came with complaints of fever, nonproductive cough, and sore throat with grade III-IV shortness of breath. Laboratory investigations revealed hypokalemia and isolates of *Burkholderia pseudomallei* on blood culture and sensitivity. High-resolution computed tomography (HRCT) of the chest showed widespread, variable-sized nodules with central cavitations diffusely scattered in bilateral lungs.

## Introduction

Melioidosis is caused by *Burkholderia pseudomallei*, gram-negative, oxidase-positive, free-living bacteria found on soil and water surfaces. This disease is also known as Whitmore's disease, as it was first recognized by Whitmore and Krishnaswami in Burma in 1912. The mode of infection is direct skin contact through percutaneous inoculation [1]. The incubation period is up to 21 days. It usually occurs in adults with immunosuppression, like diabetes mellitus, chronic kidney disease, and cystic fibrosis [2]. This disease can affect almost every organ and has a wide range of manifestations, ranging from asymptomatic infection to life-threatening multisystem involvement. It can also eventually be lethal. The lung is the most commonly affected organ, with multiorgan involvement in a large number of cases. Lung involvement may manifest as air space opacities, cavitary lesions, and numerous nodules. The most common extrapulmonary organs involved are the spleen and liver, respectively, and they typically show honeycomb-patterned abscesses. Osteomyelitis and septic arthritis are also seen when the musculoskeletal system is involved. Compared to individuals with single organ involvement without pneumonia, people with pneumonia, multiple organ involvement, or septicemia have a greater mortality rate [3]. In a clinical setting, since diagnosing is nearly impossible, understanding the condition and its different radiological manifestations might help choose which diagnostic test is best to perform in order to get a diagnosis.

## Case presentation

**Figure 1:**
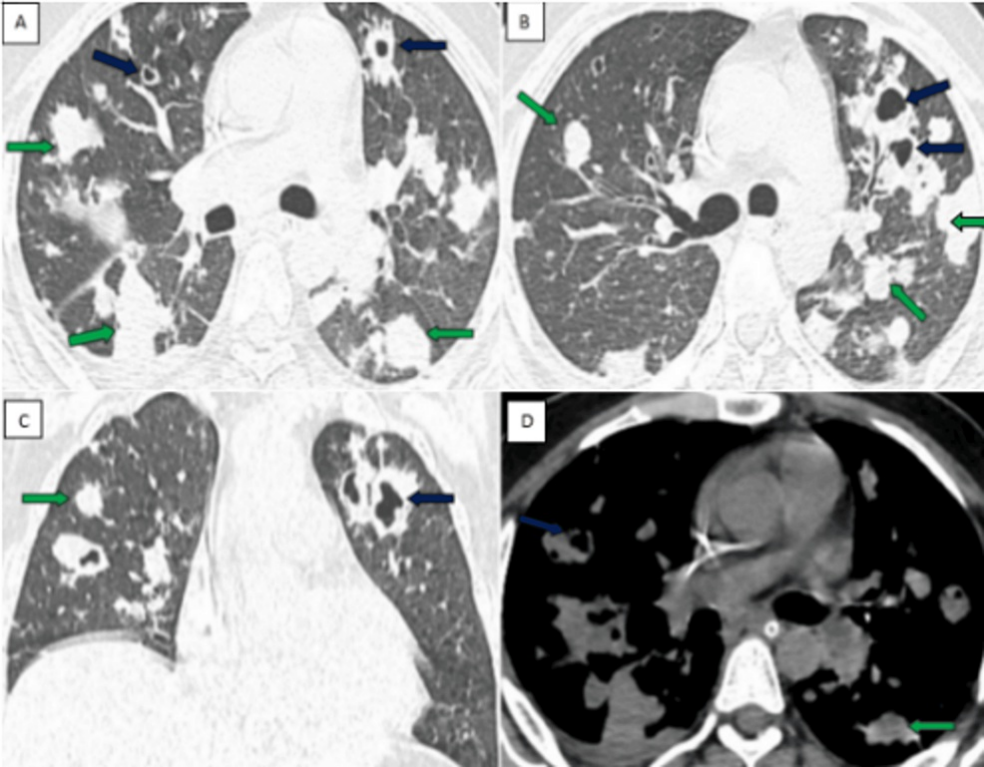
HRCT chest showing multiple nodules scattered in both lungs A and B: Axial CT chest lung window showing multiple variable-sized solids (green arrow) and cavitatory lung nodules (blue arrow) scattered in both lungs. C: Coronal image showing similar findings. D: Axial soft tissue window depicting multiple solid (green arrow) and cavitatory lung nodules (blue arrow) seen scattered in both lungs. Features suggestive of thoracic melioidosis with lung metastasis, miliary tuberculosis, and fungal infections being the close differentials

A 21-year-old male from Bidar, Karnataka, presented with complaints of nonproductive cough and grade III-IV shortness of breath for three days. History of fever and sore throat for five days. The patient is a known type II diabetic and is on medications. There was no history of nausea, vomiting, constipation, pain in the abdomen, diarrhea, or chest pain. Upon presentation to the hospital, the patient was oriented and cooperative with the following vital signs: blood pressure of 110/60 mmHg, pulse rate of 100 bpm, respiratory rate of 30/minute, and saturation of peripheral oxygen (Spo2) of 85%. On respiratory examination, bilateral air entry was present with normal breath sounds, and there was no lymphadenopathy. Routine blood investigations like CBP showed decreased hemoglobin (8.7mg/dl), RBC count (3.29 million/cu.mm), MCH (26.4 pg), MCHC (30.9 g/dl), RDW (14.1%), and platelet count (1.35 lakh/cu.mm). Serum potassium (2.8mg/dl) and magnesium (1.6mg/dl) levels were decreased. The patient was kept nil by mouth (NBM) started on 5 liters of oxygen, initial empirical antibiotic therapy with intravenous (IV) Doxycycline 100 mg, Metronidazole 500mg, and other supportive medications. A chest radiograph showed ill-defined homogenous opacities in bilateral lung fields. HRCT chest revealed multiple variable-sized nodules with central cavitations diffusely scattered in bilateral lungs (Figures 1A-1D). Based on these chest imaging findings, the initial differential diagnosis of pulmonary tuberculosis, pulmonary metastasis, and other infective etiologies was given. Diagnostic workups for pulmonary tuberculosis (bronchoalveolar lavage for AFB and GeneXpert MTB) and pulmonary metastasis (PET-CT) were negative. Samples were sent for blood culture and sensitivity, which revealed isolates of Burkholderia pseudomallei within three days, which established the diagnosis of thoracic melioidosis. Based on blood culture and sensitivity reports, the patient was started on specific intravenous antibiotics (Ceftazidime 2gm, Avibactem 0.5gm, Meropenem, and Sulbactum 1.5gm) and was continued for two weeks. Serum potassium and magnesium levels were corrected with intravenous KCl 80 meq in 500ml of normal saline (NS) over five hours (100ml/hr), intravenous magnesium 2gm in 500ml of NS stat, followed by 1gm in 100ml of NS, and monitoring the levels regularly. With the above management, the patient’s oxygen saturation (SpO2) slowly improved, requiring no oxygen support; SOB, abdominal distension, guarding, and rigidity decreased; and normal serum potassium and magnesium levels were achieved. The patient was discharged on room air with oral antibiotics (Tablet Trimethoprim 160mg, Sulfamethoxazole 800 mg, and Tablet Doxycycline 100mg) for 12 weeks, and the patient showed symptomatic improvement on follow-up visits.

## Discussion

Different stages of melioidosis may have different presentations; in acute melioidosis, multiple nodular lesions scattering in both the lungs is the most common appearance, representing hematogenous spread. The chest X-ray shows tiny nodular opacities, which may coalesce to form large nodules and may even cavitate. Focal airspace opacities (lobar, segmental, or non-segmental) are noted most commonly in the upper lobes. Abscess formation with cavitations may indicate rapid disease progression and end up with normal or mild residual fibrosis [4].

Subacute form usually presents with atypical patterns of pulmonary infiltration, such as nodules and air space opacities with or without cavitations involving the upper lobes predominantly.

The most common presentation is, however, localized upper lobe lobar infiltrates with cavity formation, making it difficult to differentiate it from tuberculosis. Lymphadenopathy and effusion formation are rare. Chronic form shows fibro-nodular lesions associated with linear densities, and cavitations can be seen. The radiographic appearance of this stage mimics pulmonary tuberculosis reactivation. After 3-6 months of treatment, the lungs can be cleared with no calcifications and little scarring, allowing for a retrospective diagnosis of melioidosis. Pericardial involvement can be seen with acute, subacute, and chronic diseases.

Dhiensiri TU et al. reported in a study that the most common finding was disseminated nodular lesions in both lungs in acute septicemia, and the next most frequent finding in this group was alveolar infiltrates, which appeared as lobar, segmental, or non-segmental focal consolidations and were usually in the upper lobes with no cavities. The chronic form was characterized by a mixed infiltrate with cavities [5].

Radiological mimics of thoracic melioidosis

The radiological findings of thoracic melioidosis are similar to those of pneumonia caused by other germs in the acute stage. The imaging findings in chronic melioidosis resemble chronic cavitary lung diseases, tuberculosis, aspergillosis, blastomycosis, histoplasmosis, coccidioidomycosis, paracoccidioidomycosis, noninfective bronchogenic carcinoma, and lung metastasis [6]. In pulmonary tuberculosis, the lesions are uniform or discrete nodular lesions with lymphadenopathy and show apical fibrosis and granuloma formation (caseous) during the healing process. Whereas melioidosis shows multiple nodular lesions scattering in both the lungs, which may coalesce to form large nodules and may even cavitate with upper lobe predominance. Lymphadenopathy is rare in melioidosis and less apical fibrosis, and usually, no granuloma forms during the healing process. A confirmatory diagnosis is done by CB-NAAT. Findings indicative of fungal infection include a single cavitating or several cavitating opacities or masses in the non-dependent cavity that have a crescent-shaped air collection [7]. Lung metastasis shows well-circumscribed, rounded lesions more often in the periphery of the lung. Prominent vessel heading (Feeding Vessel Sign) to lesions also gives a clue for diagnosis. A clinical history of weight loss and the primary source of malignancy may be helpful. A confirmatory diagnosis of lung metastasis can be done by PET-CT.

## Conclusions

Thoracic melioidosis is truly a great masquerader and mimicker of other common infectious conditions and even lung metastasis. The diagnosis of thoracic melioidosis is to be included in the differential diagnosis of multiple cavitary lung nodules in an appropriate clinical setting. However, blood culture remains the ultimate confirmatory test for the diagnosis of melioidosis. In this case, the differential diagnosis of multiple cavitary lung nodules was lung metastasis, tuberculosis, and other infections. A later diagnosis of melioidosis was made through the blood culture. Hence, knowledge of these imaging findings of multiple cavitary pulmonary nodules in thoracic melioidosis should also be sought as a differential diagnosis in a proper clinical scenario.
